# Software for Brain Network Simulations: A Comparative Study

**DOI:** 10.3389/fninf.2017.00046

**Published:** 2017-07-20

**Authors:** Ruben A. Tikidji-Hamburyan, Vikram Narayana, Zeki Bozkus, Tarek A. El-Ghazawi

**Affiliations:** ^1^School of Engineering and Applied Science, George Washington University, Washington, DC, United States; ^2^Computer Engineering Department, Kadir Has University, Istanbul, Turkey

**Keywords:** computational neuroscience, brain network simulators, spiking neural networks, comparative study, phenomenological model, conductance-based model

## Abstract

Numerical simulations of brain networks are a critical part of our efforts in understanding brain functions under pathological and normal conditions. For several decades, the community has developed many software packages and simulators to accelerate research in computational neuroscience. In this article, we select the three most popular simulators, as determined by the number of models in the ModelDB database, such as NEURON, GENESIS, and BRIAN, and perform an independent evaluation of these simulators. In addition, we study NEST, one of the lead simulators of the Human Brain Project. First, we study them based on one of the most important characteristics, the range of supported models. Our investigation reveals that brain network simulators may be biased toward supporting a specific set of models. However, all simulators tend to expand the supported range of models by providing a universal environment for the computational study of individual neurons and brain networks. Next, our investigations on the characteristics of computational architecture and efficiency indicate that all simulators compile the most computationally intensive procedures into binary code, with the aim of maximizing their computational performance. However, not all simulators provide the simplest method for module development and/or guarantee efficient binary code. Third, a study of their amenability for high-performance computing reveals that NEST can almost transparently map an existing model on a cluster or multicore computer, while NEURON requires code modification if the model developed for a single computer has to be mapped on a computational cluster. Interestingly, parallelization is the weakest characteristic of BRIAN, which provides no support for cluster computations and limited support for multicore computers. Fourth, we identify the level of user support and frequency of usage for all simulators. Finally, we carry out an evaluation using two case studies: a large network with simplified neural and synaptic models and a small network with detailed models. These two case studies allow us to avoid any bias toward a particular software package. The results indicate that BRIAN provides the most concise language for both cases considered. Furthermore, as expected, NEST mostly favors large network models, while NEURON is better suited for detailed models. Overall, the case studies reinforce our general observation that simulators have a bias in the computational performance toward specific types of the brain network models.

## 1 Introduction

Substantial progress of experimental methods in neuroscience is leading to an increasing amount of experimental data becoming available for theoretical and computational analyses. Simulations of quantitative models of individual neurons and networks are the critical component for understanding brain function under normal and pathological conditions. Indeed, computational neuroscience plays a critical role in the study of many neurological disorders, including epilepsy (Soltesz and Staley, 2008). Theoretical and computational studies of brain networks are a critical part of both the US BRAIN initiative (Insel et al., 2013) and the European Human Brain Project (Markram, 2012). Moreover, due to their ever increasing complexity, modern neural models demand extensive computational power, which require integration with high-performance computing (HPC). To meet the needs of the community, researchers have developed and released several software packages that are suitable for HPC platforms and provided online access to HPC resources for these packages (see, for example, the Neuroscience Gateway portal, Sivagnanam et al., 2013).

Currently, there are many software packages for brain network simulations that are available as open-source computer programs and can be downloaded free of charge. Moreover, users have a wide choice of tools (software “front-ends”), which enable users to describe their models in a unified format, allowing them to switch freely from one simulator to another without redesigning their models. For example, researchers can develop models in the Python language (PyNN; Davison et al., 2009) or through a graphical interface (neuroConstruct; Gleeson et al., 2007). This has contributed to a significant growth in the development of universal descriptions for neurons, synapses, and connections, which can potentially be ported to any of the network simulators (see for example, NeuroML, Gleeson et al., 2010).

Although software front-ends can hide the implementation complexity of the underlying neuron models used in their network, the most common model development cycle in computational neuroscience research still involves direct development and simulation on a specific simulator, but not over front-ends. Considering all these issues, the critical features that are important in brain simulators are

1. computational performance,
2. code complexity for describing neuron models,
3. user interface and user support, and
4. integration with parallel HPC platforms.

The authors of some of the most popular brain simulators and front-ends published a review in 2007 of their respective software packages that included a detailed comparison of the software (Brette et al., 2007). However, independent evaluation of brain networks simulators has not been done yet.

This article seeks to carry out an independent evaluation of some of the popular brain networks simulators from the perspective of a computational neuroscience researcher and deliver detailed comparisons of the software based on the four critical features listed above. In our evaluation, we study and compare four software packages in detail. Three of the simulators are the most popular packages in use, as evidenced from the total number of records in *Model DB* (Hines et al., 2004)—*NEURON* (Carnevale and Hines, 2006), *GENESIS* (Bower and Beeman, 1998), and *BRIAN* (Goodman and Brette, 2009). Note that in the original publications the authors capitalize only the first letter of the software name for Brian; however, we will use all uppercase letters in BRIAN to indicate that we are referring to the software’s name. We added the *NEST* simulator (Gewaltig and Diesmann, 2007) to these three, which is a flagship simulator of the Human Brain Project (personal communication T.A.E.-G. with Marc-Oliver Gewaltig). Based on the user documentation, a literature search, and a source code examination, we estimate several critical characteristics of the selected software: the range of models, simulation ambit; computational architecture; computational efficiency; tools for model parallelization; and usability and support for users. We used the *Model DB* website to define the usage frequency for each of the four software being compared over the last 3 years. Finally, we performed an analysis on two case studies using each piece of software; one for a large network with simple neural and synaptic models and the other for a smaller network with more sophisticated models. The two case studies were selected to represent the most extreme cases in the model-type domain, which can be simulated on the selected software. The main purpose of performing two case studies is to prevent a bias in the comparison as these different types of models require different characteristics of the software package.

## 2 Materials and Methods

### 2.1 Selection of Most Popular Software

To identify the three most popular software packages, we analyzed the “simulator” page at *Model DB* website (https://senselab.med.yale.edu/ModelDB/FindBySimulator.cshtml). We removed all general-purpose languages and software such as MATLAB, Python, C/C++, XPP, and Mathematica, from the list. Of the software remaining, the three most popular simulators, *viz*, NEURON, GENESIS, and BRIAN, were thus selected for further analysis.

### 2.2 Software Characterization

First, we selected the model range used in simulations of Brain Networks in the literature (Koch and Segev, 1998; Dayan and Abbott, 2001; Gerstner and Kistler, 2002) as well as in recent reviews (Brette et al., 2007). We also used examples from the *Model DB* website and studied the source code to identify the range of neural and synaptic models that can be realized using each software package. A literature search and browsing of documentation/tutorials was used to characterize the computational architecture for each software. We studied the available source code and literature for each package to define the potential computational efficiency and tools for model parallelization. An examination of the user documentation and software websites allowed us to estimate the software package usability and support for users. Finally, we estimated the dynamics of simulator usage by the community in two interdependent ways. We queried the *Model DB* website for models implemented in each simulator and filtered the results to obtain the number of public records in *Model DB* over the last 3 years. We also used official NEURON and NEST websites to obtain the number of publications in which these simulators were used. Unfortunately, the GENESIS website does not track publications after September 2007, and the BRIAN website does not track publications, which use BRIAN at all. We obtained rough estimates of the number of publications, which exploit these simulators by querying the number of citations for (Bower and Beeman, 1998, The Book of GENESIS) and two key BRIAN publications (Goodman and Brette, 2008, 2009) in Google-Scholar. Note that we did not exclude self-citations and “technical” publications from citation indexes; therefore, these indexes may be overestimated.

We then performed two case studies using each simulator apart from GENESIS, to confirm our estimations. GENESIS was not included in the comparison because we did not find a valuable example of a module for the leaky integrate-and-fire model or an approach to organize a robust Python interface with GENESIS (probably due to our limited experience with this simulator).

#### 2.2.1 Case Study 1

Classical Pyramidal InterNeuron Gamma (PING) network (Brunel and Wang, 2003; Atallah and Scanziani, 2009) was used as an example of a network with simple individual neurons. The network consists of 5,000 standard leaky integrate-and-fire (LIF) neurons randomly connected by delta synapses with constant axonal delay. In the network, 4,000 (80%) neurons were assigned as excitatory and 1,000 (20%) as inhibitory neurons. The evolution of membrane potential of each neuron in the population is described by a first-order differential equation with resetting:

(1) $$\frac{dv}{dt}=-\frac{v}{\tau }+\sum_{j}\;g\delta (t-t_{j}^{\prime }-d);\;v>\theta :v=v_{r},\;t^{\prime }=t$$

where *θ* is a threshold; $t_{j}^{\prime }$ is the time of spike of the *j*th presynaptic neuron, *τ* is a time constant of membrane potential; *g* is the synaptic weight; *δ*() is the Dirac’s delta function, *d* is the axonal delay, and *v_r_* is a reset membrane potential. For simplicity, in this case study, we set the threshold to *θ* = 1 mV, the reset voltage and time constant were set to *v_r_* = 0 mV, *τ* = 10 ms, correspondingly. The next spike for any neuron in both populations could not be generated during the refractory period after a previous spike. The refractory period was set to 5.01 ms.

In addition to 5,000 LIF neurons, the network consists of 500 Poisson spike generators. Connection probabilities were set up as follows:

- inside the excitatory population, *P_ee_* = 0.005;
- from excitatory to inhibitory populations, *P_ei_* = 0.3
- inside the inhibitory population, *P_ii_* = 0.3;
- from inhibitory to excitatory population, *P_ie_* = 0.2; and
- from Poisson spike generators to excitatory neurons, *P_se_* = 0.15.

Synaptic weights (*g*) and delays (*d*) for these connections were set to *g_ee_* = *g_ei_* = 0.009 mV, *d_ee_* = *d_ei_* = 0.8 ms; *g_ii_* = *g_ie_* = −0.05 mV, *d_ii_* = *d_ie_* = 2.1 ms; and *g_se_* = 0.025 mV, *d_se_* = 0.5 ms. Each Poisson spike generator produced a random spike sequence with 50 Hz mean frequency. A connectivity pattern as well as Poisson generator spike sequences were generated once and were identical for this case study using all studied simulators.

The simulation was run for 1 s of model time on Linux box (CPU: dual-core Intel Core i5 2.70 GHz, RAM: 16 Gb, HDD: 512 Gb) under the operating system Linux Mint 18.1 KDE edition. The network was implemented as Python scripts for each software. Python’s standard library function *time* was used to define the time required to build a network and time required to simulate 1 s of network dynamics. We measured *building time* as the difference between the time value just prior to a first call of the first simulator’s function and the time value when the simulator was ready to run the simulation. The *simulation time* is the difference between the time just before calling the simulation’s function to run the simulation and just after simulator returns back control to the Python script. In this study, we used BRIAN version 2.0.1, NEURON Release 7.4 (1370:16a7055d4a86) 2015-11-09 and NEST 2.12.0. NEST and NEURON were compiled locally from source code, and BRIAN was obtained *via* pip interface to PyPi. The scripts do not control for the numerical methods to solve differential equations, apart from BRIAN, which required set numerical methods explicitly. The script sets recommended a semianalytical “linear method” for BRIAN, which worked in default “numpy” regime (see Discussion for more details). Here, we specifically use the software in default mode: “off-the-shelf,” which is an exponential-Euler solver for NEST and NEURON. The scripts control the time step for the solution, which was set to 0.1 ms for all simulators. To estimate peak memory usage, we dumped the output of the *top* command every second and report the maximal memory allocation in a Figure S1 in Supplementary Material.

#### 2.2.2 Case Study 2

A recently published PostinhIbitory Rebound—InterNeuron Gamma (PIR-ING) network (Tikidji-Hamburyan et al., 2015)—was used to study a network with complex models of individual neurons. The network consisted of 400 classical Hodgkin–Huxley (HH) (Hodgkin and Huxley, 1952) neurons with double-exponential synapses. The dynamics of each neuron is described by four dynamical variables for each neuron plus two dynamical variables for each inhibitory synapse. We also added one additional dynamical variable for exponential synapses to be able to compare BRIAN and NEURON with the standard NEST hh-module. The evolution of membrane potential and all other dynamical variables are given by standard systems of first order differential equations (2), where *g_Na_, g_K_*, and *g_L_* are the maximum conductance values for sodium, potassium, and leak currents; *E_Na_, E_K_*, and *E_L_* are the reversal potentials for the corresponding currents; *g_i_, E_i_* and *g_e_* and *E_e_* are the peak conductance and reversal potentials for inhibitory and excitatory synapses, respectively; *c* is the membrane capacitance; *α_X_*(*v*) and *β_X_*(*v*) are the standard HH rate functions for sodium activation (*X* is *m*), sodium inactivation (*X* is *h*), and potassium activation (*X* is *n*) (Hodgkin and Huxley, 1952). Each neuron was sparsely connected by inhibitory synapses with 40 random neurons within the population. For all connections, the axonal propagation delay was set to *d_i_* = 3 ms. Time constants for synapses were set to *τ_i_*_1_ = 0.99 ms and *τ_i_*_2_ = 1 ms. Inhibitory synaptic conductance and reversal potential were set to *g_i_* = 2 nS and *E_i_* = −75 mV, respectively. Excitatory synapses were not used in this case study but were added to equalize the total number of differential equations in the model.

(2) $$\begin{array}{c} \frac{dv}{dt}=[g_{Na}m^{3}h(E_{Na}-v)+g_{K}n^{4}(E_{K}-v)+g_{L}(E_{L}-v)\\ \;+I+g_{i}k_{i}(b_{i}-a_{i})(E_{i}-v)+g_{e}a_{e}(E_{e}-v)]/c\\ \frac{dm}{dt}=\alpha_{m}(v)(1-m)-\beta_{m}(v)m\\ \frac{dh}{dt}=\alpha_{h}(v)(1-h)-\beta_{h}(v)h\\ \frac{dn}{dt}=\alpha_{n}(v)(1-n)-\beta_{n}(v)n\\ \frac{da_{i}}{dt}=-\frac{a_{i}}{\tau_{i1}}+\sum_{j}\;g_{i}\delta (t-t_{ij}^{\prime }-d_{i})\\ \frac{db_{i}}{dt}=-\frac{b_{i}}{\tau_{i2}}+\sum_{j}\;g_{i}\delta (t-t_{ij}^{\prime }-d_{i})\\ \frac{da_{e}}{dt}=-\frac{a_{e}}{\tau_{e}}+\sum_{j}\;g_{e}\delta (t-t_{ej}^{\prime }-d_{e}) \end{array}$$

The network was implemented using Python scripts for each simulator. The simulation was run for 500 ms of model time with the same software and hardware setup as described in Case Study 1. A similar procedure for evaluating the building time and simulation time was used. As in Case Study 1, the script controlled only the time step, which was set to 0.05 ms for each simulator. In the second case study, “off-the-shelf” default numerical solvers were as follows: Runge–Kutta 4/5 implemented in the GNU scientific library for NEST and exponential Euler for ion channels and a modified implicit Crank–Nicholson method for voltage (Hines and Carnevale, 1997) for NEURON. For BRIAN, the recommended “linear solver” cannot be applied due to the problem complexity of the neuronal model. We tested the “exponential-Euler solver,” which produced a solution faster than NEURON and NEST. However, to keep the integration method accuracy closer to that of NEST and NEURON, we use the Runge–Kutta (rk4) solver. Therefore, note that the *simulation time*, reported in this Case Study for BRIAN, can be reduced to less than 3 s, if the “exponential-Euler solver” is used.

### 2.3 Estimation of Code Complexity for Both Case Studies

We used source code from the main stable branch of NEURON and NEST to study the complexity of modules used in both case studies. For Case Study 1, NEURON module intfire1.mod and NEST module iaf_psc_delta.h iaf_psc_delta.cpp were used for a code complexity study. In Case Study 2, NEURON modules exp2syn.mod, expsyn.mod, hh.mod, and NEST module hh_psc_alpha.cpp hh_psc_alpha.h were examined. Note that we include Python scripts in the code complexity estimation. For any module used, comments, empty lines, and unnecessary spaces were removed. We then counted the number of lines as well as number of characters, which a potential user has to write as code if they decide to develop a similar module “from scratch.” It should be noted that recently developed NESTML (Plotnikov et al., 2016) may significantly decrease code complexity of NEST modules in future. For BRIAN, all mathematical expressions were included in a single Python script, which was used to define the code complexity. We used examples from the BRIAN tutorial and user documentation to develop the most “authentic” code for BRIAN.

#### 2.3.1 Source Code Sharing

The source code of the models and required scripts will be made publicly available *via* the ModelDB website after publication of this article: http://senselab.med.yale.edu/ModelDB/showModel.cshtml?model=222725.

## 3 Results

### 3.1 Determining the Most Popular Software for Brain Network Simulations

**Figure 1:**
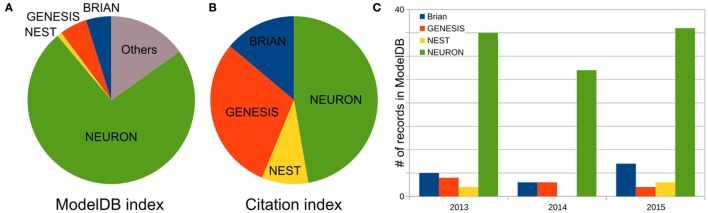
Frequency of software usage. **(A)** Total number of public records for three top most popular simulators and NEST in the *ModelBD*. Full list of considered software in Others and number of records in the *ModelBD* are given in the Table S1 in Supplementary Material. **(B)** Number of citations for each selected software. For NEURON and NEST, the numbers of citations are taken from official website. For GENESIS and BRIAN, this number was obtained as a number of articles which cite a key publication(s) of each simulator (see main text, Materials and Methods section, for more details). **(C)** Number of records in the *ModelDB* for each selected software published in the last 3 years.

As the first step, we defined the three most popular software for brain network simulations. As described in Materials and Methods, we estimated each simulator’s usage by the number of records publicly available on the *Model DB* website. We excluded any general-purpose computer languages from consideration. Figure 1A shows the relationship between the total number of records for the top three packages: NEURON (73.7%), (p)GENESIS (5.3%), and BRIAN (4.9%), as well as for one additional package NEST (0.9%) in *Model DB*. These four packages are characterized below. Note that we do not exclude front-end packages from the “others” category (15.3%), which supports our finding that the development of models for specific simulators is a dominating paradigm in the computational neuroscience field. A full list of simulators and number of records in Model DB is given in Table S1 in Supplementary Material.

### 3.2 General Characteristics for Brain Network Simulators

We referred to the user documentation, results from a literature search and source code to examine the most crucial characteristics of the selected software: model range and limits of implementation of each simulator; computational architecture, efficiency and tools for model parallelization, and program usability and support.

#### 3.2.1 The Range of the Model of Each Software Package

One of the most critical characteristics of software for simulating brain networks is the model range that defines the envelope for computation. Researchers use a wide range of models to study brain neural networks: from “dynamicless” stochastic models to detailed 3D morphological models with an accurate representation of spatial-temporal integration in dendrites, modeling extracellular currents around dendrites and cell body, and modeling of individual molecules in intracellular signaling pathways. Moreover, while some of these models can be implemented without writing any scripts by using a Graphical User Interface (GUI), other models need to be coded in some specific or general-purpose computer language. Although modeled processes have simple intuitive explanations, for example, diffusion or voltage propagation in dendrites, the mathematical and computational implementation is not simple and requires extensive knowledge of partial differential equations and numerical methods. Therefore, implementation of such models may be a challenge for neuroscientists.

In Table 1, we consider a range of models from dynamicless to a highly accurate representation of spatial-temporal integration and chemical diffusion–reaction. Depending upon the scale of neural tissue organization at which a network is studied and the availability of computational resources, the researcher may want to use simplified models of individual neurons, synapses, or network structure. For example, if the main purpose of a model is to study network mechanisms (see Section 1, for example, or Atallah and Scanziani, 2009 as a real-life example), an accurate representation of the dynamics of individual neurons may be beyond the scope of such research; therefore, neurons and, possibly, synapses can be represented by simplified or even “dynamicless” models. In contrast, if the study of spatial-temporal integration in non-linear dendrites is the research question (see Mainen and Sejnowski (1996) and Jarsky et al. (2005) as outstanding examples), a network, in which the neuron is embedded, may be presented as a simplified model or totally ignored. In this case, the electrical properties of membrane excitability along a dendrite, chemical kinetics, chemical diffusion of ions inside of the neuron, as well as electrical currents in extracellular media must be modeled with great detail. If computational resources are limited, a compromise between the accuracy of neurons, synapses, network representation, and the time required to compute an evolution of network dynamics needs to be found. In Case Study 2 (see Section 3.3.2), we use an example in which the interaction between the dynamics of individual neurons and network dynamics is critical for the studied phenomenon. In similar cases, researchers may have to reduce both the network size and details of neuron and synapse representation. Indeed, single neurons may be presented in some cases as two- or single-compartment models but with representation of cross-membrane non-linear currents; see Mainen and Sejnowski (1996) as representative examples of such a reduction. However, if the accuracy of representation is critical for the studied phenomena (see Blue Brain and Human Brain Projects, Markram, 2006, 2012, as a most impressive example), both the non-linear spatial-temporal integration in individual neurons and the fully detailed networks should be modeled. Therefore, the ability of a simulator to utilize high-performance computing is critical in these cases (see Section 3.3.2 for details). For each software under study, we consider a generic way of implementing each model type in the range discussed above. The implementation may be achieved using GUI, writing modules, and modification of existing modules or cannot be achieved at all. We characterize each simulator separately later.

**Table 1:** Neural models range.

	NEURON	NEST	BRIAN	GENESIS
Neuron model without dynamics	M	M	Y	M
Neuron model with simplified and discontinuous dynamics *Examples: Leaky Integrate-and-Fire (LIF), Izhikevich or Quadratic LIF; Exponent Leaky Integrate-and-Fire (eLIF)*	M	M	Y	M
Neuron model with simplified and continues dynamics *Examples: FitzHugh–Nagumo, Morris–Lecar*	M	M	Y	M
Single compartment, conductance-based model—temporal integration (point neuron) *Examples: Single-Compartment Hodgkin–Huxley model*	YG	M	Y	Y
Can conductance-based descriptions of ion channels be added to the neuron model? *Example: h-channel*	YG/M	m	Y	M
Neuron model with simplify morphology (2-compartment model) *Example: Pinsky–Rinzel model*	YG	M	Y	M
Full spatial reconstruction of individual neuron morphology cable property spatial-temporal integration (multicompartment model) *Example: Mainen–Sejnowski model*	YG	M:E	Y	Y
Extracellular/intracellular chemical kinetics *Example: Ca^2^*^+^ *dynamics*	M	m	Y	Y
Can new ion be added to existing model	YG/M	m	Y	M
Radial diffusion	M	M:E	Y	Y
Longitudinal diffusion	M	M:E	N	N
Currents in external medium *Examples: to model transcranial magnetic stimulation or deep brain stimulation*	M	N	Y:E	M:E
New model of chemical synapse	M	m	Y	M
New model of electrical synapse	M	m	Y	M
New model of learning rule	m	M	Y	M

*YG can be done in GUI without programming; Y can be done in script without writing modules; N cannot be done; M can be implemented through external modules; Y:E or M:E can be implemented in script or through external modules but requires extensive additional knowledge outside of the presumed user ability, such as numerical methods, physics, and partial differential equations (in some sense Y:E ≈ M:E ≈ N); and m cannot be implemented as an independent module but may be done through code modification*.

In general, all simulators can support “dynamicless” neuron models. For example, it is possible to use selected software for implementation of an artificial neural network with the perceptrons or a simulating annealing algorithm for energy minimization. It is very artificial to implement such a model on software developed for dynamic systems, i.e., representing a one-step energy evaluation as a time step of a dynamic system. Therefore, we consider this kind of model as a boundary for Brain Networks simulators, beyond which software for artificial intelligence is a better choice. In NEURON, NEST, and GENESIS, these kinds of models can be implemented as external modules. In BRIAN, such models can be implemented as iterative variables. We did not find any examples of realizing these for GENESIS and BRIAN, but we only relied on the software documentation.

Models with discontinuous dynamics are at the minimum complexity level for Brain Networks simulators, for example, classical leaky integrate-and-fire (LIF) (Koch and Segev, 1998), Izhikevich model (Izhikevich, 2003) or Quadratic LIF (qLIF) (Ermentrout, 1996), Exponential LIF (eLIF) (Brette and Gerstner, 2005), and many others. Again, all simulators can support this kind of model. All simulators, except BRIAN, require the development of an external module. We did not find any examples of this kind of model for GENESIS, even though documentation indicates that such modules may be developed.

The next class of models is characterized by continuous dynamics, while the biophysical nature of temporal integration is extremely simplified. Such models help understand the general dynamics of membrane potential as well as different types of neuron excitability (Rinzel and Ermentrout, 1998). Classical examples of such models are FitzHugh–Nagumo (FH–N) (FitzHugh, 1961) or Morris–Lecar (M–L) (Morris and Lecar, 1981) models. Again all simulators can support this class of models.

The point model, also known as a single-compartment model, aims to model biophysical processes of membrane potential dynamics accurately. This includes cross-membrane currents, membrane capacitance, etc., but without modeling their spatial integration. Such models are a subclass of so-called “conductance-based” models. In conductance-based models, each cross-membrane current is represented as a non-linear conductance, which is connected in series to a battery with voltage equal to Nernst’s reversal potential. The conductance is a complex dynamic model with one or more dynamic variables. Therefore, this class of model is very big, due to many possible combinations of ion channels in different neurons. The classical example of a single-compartment conductance-based model is the Hodgkin–Huxley model (Hodgkin and Huxley, 1952). All simulators are able to support this kind of model. Note that NEURON allows the development of single- and multicompartment conductance-based models in GUI and does not require any coding, including adding new mechanisms (modules); therefore, NEURON is widely used as a toolkit for educational purposes.

A researcher may want to add a new channel and update an existing model. Different simulators exploit different paradigms to achieve this. In NEURON, the user needs to develop a new module and set up the distribution of the new channel conductance along a neuron body. Note that module realization and model modification may be done through GUI, but in practice, researchers prefer to develop the code. In NEST, modification of the whole neuron model is the only option. It may be difficult for an inexperienced user if the model is more complicated than a just a few channels. For example, implementation of the McCormick two-compartment model of a cortical pyramidal cell will consist of at least 10 ion channels with 16 activation/inactivation variables, each of which has two rate functions, plus calcium and sodium dynamics, and synaptic dynamics, which results in relatively massive code. Such a code would require using a structural approach and having good skills in programming. BRIAN exhibits the same problem as NEST, because modularity is not an intrinsic property of BRIAN. Finally, GENESIS can support any ion channel, which may be implemented as one of the embedded model classes without developing a specific module. For example, if an ion channel can be described by two voltage-dependent gating variables and one calcium-dependent variable with Boltzmann rate functions, such a channel can be implemented by just setting a required coefficient for the embedded model. Moreover, precalculated lookup tables (see below) theoretically allow for defining arbitrary voltage-dependent rate functions in the model. However, if new channel dynamics cannot be fit to any pre-installed models, the user has to develop an external module in C.

Two-compartment models can be considered as an intermediate model class between point neural models and a full spatial reconstruction of neuron morphology (see for example, Pinsky and Rinzel, 1994; Destexhe et al., 1996). In such models, spatial integration is represented by coupled point models (compartments) with different ion channels in each compartment. Usually, one compartment represents an axon or/and soma of a neuron and is called the axo-somatic compartment. Another compartment represents the dendrites. The strength of electrical coupling between the compartments and their size may be used to mimic different neuron morphologies (Mainen and Sejnowski, 1996). Indeed, all simulators can support such models. For NEURON and GENESIS, these kinds of models should be implemented through the same mechanism as multicompartment models. In NEST, these models are collapsed into one module with different synaptic inputs for each compartment. In BRIAN, the user can use both cable model objects or hold all required equations in one neuron object.

The most accurate modeling of signal processing in individual neurons requires reconstruction of neuron morphology as well as the distribution of ion channels along dendrite trees. A classical model for dendrites is a non-linear cable, which is described by a partial differential equation (Dayan and Abbott, 2001). This class of model is considered as a “native” model for NEURON and GENESIS. BRIAN can support this kind of model through a set of morphology objects, such as a soma, cable cylinder, and cable segment. An example of a fully reconstructed neuron can be found among the examples included with BRIAN. In NEST, the user may develop a module for full dendrite tree reconstruction, in theory. However, the realization of such a model requires an extensive knowledge in numerical methods, good skills in programming in C++, as well as a tremendous amount of effort. Although a template for the non-linear cable model recently appeared in the NEST development repository,^1^ full reconstruction of a neuron morphology using this template is still a serious challenge, which is unlikely to be met by a researcher with a *Neuroscience* background.

A cross-membrane electrical current may depend upon inner and outer ion concentrations. A classical example of such a dependency is a calcium-dependent potassium current, where conductance is a function of the intracellular calcium concentration. In simple models, calcium kinetics is usually defined as a first-order ordinary differential equation (ODE), which is easy to embed into a single- or two-compartment model. However, the dynamics of calcium concentration is much more complex than a first-order ODE in a real neuron. Calcium may be buffered by calmoduline and many other molecules, pumped into or released from mitochondria and endoplasmic reticula. Calcium ions can diffuse inside a neuron both radially and along dendrites (longitudinal diffusion). This turns a dendrite model into a non-linear diffusion–reaction system. NEURON and GENESIS can fully support the complex intracellular diffusion–reaction system. A user can even add new ions to the system. GENESIS, however, cannot support longitudinal diffusion. For NEST, the user can develop a specific module or modify existing ones for the introduction of a complex model of chemical kinetics and diffusion. However, it is very challenging for a neuroscientist and, probably, could not be done in the time scale required for model development. In BRIAN, chemical kinetics as well as radial diffusion can be modeled by adding additional dynamical variables to an equation set, but again, an accurate realization will require additional knowledge in numerical methods. We did not find any evidence that the BRIAN section module can support longitudinal diffusion.

During electrical stimulation of a brain in some pathological cases (for example, in deep brain stimulation therapy), currents in extracellular media play a critical role in the stabilization of neuron activity. In addition, consideration of an extracellular solution as a “ground wire” is not a very accurate model. Therefore, the modeling of currents in extracellular media may improve the accuracy of a model and may also be critical for some applications. We found that only NEURON can model extracellular currents off-the-shelf. While this is possible in BRIAN and GENESIS due to the embedded geometry in the compartment module, it requires extensive knowledge far beyond the *Neuroscience* field. In NEST, individual neurons are considered mostly as point processes without geometrical representation; therefore, it seems very unlikely that modeling of extracellular currents is possible in this simulator.

Finally, the user is able to add a new model of chemical and electrical synapses as well as the learning rule in all the studied simulators. However, it should be noted that the difference between developing an independent module for synapses (M) and modification of existing modules for each neuron (m) may significantly increase the amount of programming work for a potential user. For example, if there are five models of neurons in a network and a new synaptic model needs to be added to all the neurons, in NEURON, it would be done by developing a module and creating a new synaptic object, while in NEST, the user would need to add a stereotypic code to all five modules. Note that the NEST programming architecture requires implementation of a synaptic model in a neuron class through a specific connector handle. Such an architecture prevents simple realization of new models for all neurons or even automatic addition of new models during a preprocessing compilation stage.

In summary, our model range study shows that brain network simulators may have a bias toward some specific models. For example, NEST is mostly designed for modeling large networks with simple individual neurons, while NEURON and GENESIS mostly focus on the reconstruction of spatial-temporal integration in non-linear dendrites. However, all simulators tend to expand specific model ranges and provide a universal environment for computational study both of individual neurons and brain networks. At this moment, NEURON supports the widest range of the models.

#### 3.2.2 Computational Architecture, Efficiency, and High-Performance Computing Utilization

The organization of computation is an important property of a brain network simulator. There are several levels of computational organization, which are totally or partially transparent for users. Such levels are not independent and constitute a “computational architecture” for a particular simulator. In Table 2, we consider three main levels of computational architecture for each studied software. To avoid confusion, it should be mentioned that brain neural networks consist of several levels of complexity: interneuron connectivity, morphology of individual neurons as a basis for spatial-temporal integration, and processes on small membrane loci. Not surprisingly, the layers of computational architecture follow the natural structure of the biological neural networks. Therefore, there is a top layer of network organization, a middle layer of neuron description, and a bottom layer of minimum module computation.

**Table 2:** Computational architecture.

	NEURON	NEST	BRIAN	GENESIS
Language for network description	SLI: NL(HOC) or Py	SLI: NL(SLI) or Py	SLI: Python	SLI: NL(G) or Py
Language for neuron description	SLI: NL(HOC) or Py	Compiler: C++	SLI in Py	SLI: NL Compiler: C
	Compiler: NL(NMODL)			
Languages for modules	Compiler: NL(NMODL)	Compiler: C++	Compiler NL In-line compilation	Compiler: C

*SLI, script language interpreter; NL, native, software specific language; Py, Python. Note that NEST native script language is also called SLI. To avoid confusion, we mark the names of native languages as NL (Language name)*.

As showed in Muller et al. (2015), Python plays an important role in modeling neural networks. All simulators tend to use Python as a second language for neural network descriptions. In NEURON, description of individual neurons and networks may be done either in a native “c-like” language (hoc), on pure Python, or in a mix of Python and hoc (Hines et al., 2009). NEST also can interpret Python instead of a native “stack-machine” language (SLI), for network description, but not for individual neurons (Gewaltig and Diesmann, 2007; Eppler et al., 2009). GENESIS can use Python as well (Cornelis et al., 2012), although the software is strongly oriented toward the development of an independent native language (G-language; Bower and Beeman, 1998). BRIAN shows the most intensive use of Python not only for networks but also for whole model description languages (Goodman and Brette, 2009). In all simulators, a native script language or Python is used for the network definition. This top level of computational architecture is processed by an interpreter, due to the assumption that network structure is a static part of any model. Indeed in many simulations, network connectivity does not change during simulation. This allows network structure to be assembled once and excludes network reconfiguration from the computationally intensive simulation of dynamics and spiking activity. Note that a static network structure may limit the application of the simulators. For example, a network is not a static structure during pre- and postnatal development (Tikidji-Hamburyan et al., 2016) or in several pathological conditions, for instance in Alzheimer’s disease.

The minimum level of complexity in computational architecture is a single computational module. A module may represent a whole neuron with all the differential equations for both neurodynamics and synaptic dynamics. However, for simulators that are more oriented toward modeling spatial-temporal integration, a module may represent: a dendrite branch with the characteristic set of differential equations for modeling neurodynamics on the local spatial locus of the membrane, or implement a mathematical model for a single ion current, ion concentration, or intra/extracellular ion diffusion. Therefore, a module has a different meaning in different simulators, which we describe in detail below.

The next level of complexity deals with individual neuron descriptions. For some simulators, this is minimal. For example, in NEST, individual neurons are considered as nodes and connections in a graph structure; therefore, each neuron represents an individual computational block or module. In NEST, each module is a C++ class, which the user has to develop for the introduction of any new model. For NEURON and GENESIS, individual neuron descriptions have an intermediate level of complexity. For phenomenological neural models with both continuous and discontinuous dynamics, such as LIF, Izhikevich model, qLIF, eLIF and FH–N, and M–L, users have to develop the module (low level in the complexity hierarchy). In NEURON, such modules should be developed in a specific “c-like” native computer languages, NMODL. The syntax of NMODL is deeply simplified, speeding up the learning process for new users without a computer science background. In GENESIS, the user has to develop the module in C, which may be a challenge for a neuroscientist.

In contrast, if the model of an individual neuron is a multicompartment model, which addresses spatial-temporal non-linear integration, in both NEURON and GENESIS, the user needs to describe the neural morphology, electrical property of the cellular membrane, and the chemical diffusion–reaction in an interpreted language (native hoc/G or Python). In this case, the morphological structure and cable property of dendrites are parsed by the interpreter at the stage of model formation. The neuron structure and connectivity are assumed to be static during the simulation of neurodynamics. In BRIAN, the user does not need to develop an external module. Instead, the user provides the required equations for a whole neural model, if it is a phenomenological model, or for the membrane electrical balance as well as chemical kinetics and diffusion, if it is a multicompartmental model. This should be done in some extension of the Python language using specific language for physics equations. BRIAN and NEURON also check the units in the equations, which reduces the possibility of mistakes in a model. Indeed, BRIAN forces a user to systematically set all the units and does not allow simulation until all units have been homogenized, while NEURON only optionally checks the units within individual modules.

Due to high computational intensity, all simulators tend to compile modules into binary executable dynamical libraries. The NEURON routine nrnivmodl converts NMODL script into comprehensive, highly computationally efficient, hardware specific c-code and uses a system compiler to compile and to link a dynamical library (*.so on linux or *.dll on windows). During model runtime, NEURON binds this library and uses modules in the main cycle of a simulation. NEST uses a similar strategy, except that the user has to develop a module in the low-level C++ language. Moreover, the user may not have enough knowledge and experience to develop an optimal code for a given mathematical equation; therefore, computational efficacy may be low. The hope of NEST developers for high optimization during module compilation may be ephemeral, due to a limited tolerance of modern compilers to the potential inefficiency of a novice users code. GENESIS offers two approaches: first if a model can be described by one of the built-in formal forms, the user needs only to set a required coefficient for the chosen model. Second, if the model cannot be described through the built-in equations, the user has to develop a new module in a low-level C language. The second option has the same disadvantage as the NEST approach. Finally, BRIAN offers several different schemes. It can convert equations online into a Python code with *numpy* mathematical routines; or into Cython code, which Cython converts into C-code and generates modules to bind to; or into low-level C++ code. In general, all of BRIAN’s converters may be adjusted to the specific hardware architecture; therefore, BRIAN can generate highly efficient code without additional effort from the user.

In summary, as we highlight in Table 3, all simulators use binary code in their efforts to use the most efficient computations. However, as we mentioned earlier, not all simulators provide the simplest way for module development or can guarantee highly efficient binary code at the end.

**Table 3:** Computational efficiency and parallelization.

	NEURON	NEST	BRIAN	GENESIS
Are code compiled into high optimized dynamical library, to achieve maximum of computational performance on given hardware?	Y	Y	Y	Y
Does simulator/module language provide routines for tabulating right-side of differential equations and speed up computations?	Y	N	N	Y
Does the architecture allow to separate models for synapses, neuron, and learning rules to optimize amount of computations?	Y	N	Y	Y
Can user add high-level scripts into the main simulation cycle (for debugging purpose as an example)?	Y	N	Y	Y

“Embarrassingly” parallel computations, example: Parameters fitting	Y	N	N	Y
Truly distributed computations through MPI for neuron-to-neuron communication	Y	Y	N	Y
Truly distributed computations through MPI for gap junctions	Y	Y	N	N
Distributed computations for complex multicompartment neuron on several clusters nodes through MPI	Y	N	N	?
Use the advantage of biological delays to hide slow node-to-node MPI communications in event-based simulations	Y	Y	N	?
Multithread parallel computation for parallelization on single node/computer	Y	Y	Y	?
	p-threads	OpenMP	OpenMP or GPU (limited)	

The next significant improvement in performance is attributed to a popularly used optimization scheme, wherein the right-hand sides of differential equations are precomputed into lookup tables. Such tables can contain steady-state values and taus for gating variables against the membrane potential, for example. During a simulation, the solver interpolates between the points taken from the lookup table instead of carrying out a real computation. In general, such an approach significantly speeds up the calculation. For example, tabulation of standard rate functions in the Hodgkin–Huxley model with a 0.1 mV step speeds up computation by at least one order of magnitude, with an error of less than 0.3%. Moreover, widely used tables with 1 mV steps require only a small array of 120–160 (double precision) float-point numbers and provide results with an error of less than 5%. However, such lookup tables need additional memory. Therefore, objects in a model usually share the tables to avoid wasting memory. Allocation of memory, generation of tables, as well as sharing tables along model’s objects, is not a simple task for a user without a computer science background, specifically in C/C++. NEURON allows the user to turn on/off the lookup tables through two commands in the NMODL script. All details are totally hidden from the user. NEST does not provide any routines for equations, right-hand side tabulations, table sharing, or linear interpolations. Therefore, the implementation of such lookup tables is a complicated task in NEST. GENESIS directly encourages users to use a lookup table (Bower and Beeman, 1998), which provides a very flexible approach. We did not find a way to use lookup tables in BRIAN.

Neural networks are highly heterogeneous. The ability of the user to specify the right amount of computation in each part of the model can significantly increase computational efficiency. All simulators except NEST allow the setting of modules for phenomenological neurons, channels, or synapses just when they are needed. The NEST architecture, which requires compiling of neural and synaptic models in one object, does not allow a reduction of the system of equations for individual objects and compels users to use the full system, unless they develop a set of modules for all possible combinations of synaptic and neural models. For example, if conductance-based neurons in a population receive excitatory NMDA and AMPA synapses as well as inhibitory GABA synapses, but not all neurons in the population have NMDA or AMPA inputs, the user has to develop at least three modules of the same conductance-based model, namely, with NMDA + AMPA, with just NMDA, and with just AMPA synapses. If only some of the neurons within a population receive an inhibitory input, this increases the number of modules, which a user has to develop to 6. Of course, they may also use only one module with NMDA + AMPA + GABA synapses and just set unused conductance to zero. However, this will not reduce the amount of computation, due to the fact that gating variables in each synaptic model have to be evaluated for every step of the simulation.

Finally, during model development, it is critical to organize some sort of break point if some model variable has crossed a physiological limit to the values. All simulators except NEST allow a script code to be added to a novice user’s code to check for such exceptions. We did not find any way to add conditional break points to the main simulation cycle in NEST; therefore, the user has to wait until the simulation stops, even when the model contains a bug.

We next study how different simulators support high-performance computations (HPC), specifically on multicore hardware and on clusters. First, we study how simulators support the most common computational tasks in model development, such as an embarrassingly parallel problem for the study of a model parameter space (Table 3, bottom part). NEURON compiled with MPI support, and parallel GENESIS (pGENESIS) can support this type of problem. Neither NEST nor BRIAN has this kind of mechanism. For both NEST and BRIAN, embarrassingly parallel problems must be organized by external procedures in addition to a simulator. If the embarrassingly parallel tasks are to be deployed to a cluster architecture, the top level procedure must use a corresponding environment (“clusterware”), which may be a challenge for neuroscientists to define.

Second, we test if a model may be distributed on a computational cluster, when neuron-to-neuron event-based communication requires cluster internode communication. All simulators except BRIAN support this kind of utilization of HPC resources. We did not find any MPI support for the BRIAN simulator, which strongly limits its application for large networks. Note that while NEST transparently maps a network on a cluster, NEURON requires the usage of specific mechanisms for connections, to enable MPI simulation (Migliore et al., 2006).

Not all communications are event-based in a real neural network. For example, electrical synapses, also called gap junctions, require constant updates at each simulation step. Such a strong connectivity requires a high rate of message exchange using the MPI system. Both NEURON and NEST allow the use of MPI for gap junctions. A recent advance in gap junction simulation on MPI clusters (Hahne et al., 2015) improves the efficiency of the NEST simulator on a cluster for a network, which contains electrical synapses.

The next critical problem appears when a single neuron specification is too detailed and requires the distribution of computation, even though it is for a single neuron. This is a hard problem, due to the high connectivity of the model. Only NEURON allows the distribution of a single neuron model on a cluster (Hines et al., 2008).

Finally, we ask whether simulators can use an MPI delay to its advantage and map axonal delays on MPI message delays (Hines and Carnevale, 2008). A simulator can continually compute neural dynamics on the period of minimal axonal delay in a biological network without synchronization with other compute-nodes within a cluster. In contrast, if the simulator can compute and exchange messages at the same time, this allows partial or total elimination of overheads related to the synchronization calculations on and data transfer between different nodes. Thus, mapping axonal delays on MPI message delays can significantly speed up calculations on clusters. It seems that only NEURON and NEST can support this useful technique. We could not evaluate this property of pGENESIS due to limited the experience of authors with this simulator.

Modern computational paradigms are biased toward many core processors, Graphical Processing Units (GPU) or a Field-Programmable Gate Array (FPGA) computation (Iyer and Tullsen, 2015). The standard version of NEURON supports only p-threads, which is not a very effective mechanism. However, recently, NEURON was ported on GPU and multicore. This port is called “CoreNEURON” due to strong limitations on the supported mechanisms and numerical solvers (Kumbhar et al., 2016). NEST can use OpenMP parallelization and is also partially ported on GPU (Kayraklioglu et al., 2015). BRIAN offers third-party projects, which export existing BRIAN models as stand-alone executables on OpenMP or GPU. However, these exports do not support BRIAN/Python language fully and impose limitations on available functionality.

#### 3.2.3 Program Usability and Support

As we mentioned in Section 1, several software front-ends such as PyNN and neuroConstruct can hide the real implementation from a user. Usability and user level support are even more important for large size models, in addition to cases when a user applies a specific simulator as the main environment for development and simulations.

First, we ask if the simulator can be used in a GUI regime. As shown in Table 4, only NEURON has an extensive GUI interface. Other simulators do not have GUI or assume to use external libraries for GUI, or support GUI during simulation only as GENESIS.

**Table 4:** Usability and support.

	NEURON	NEST	BRIAN	GENESIS
GUI support (What can be done in GUI?)	C M T R P	P	N	R P
Inter-users/users-developers communication	Online Forum	Mail-list	Mail-list Google-group	Mail-list
Online documentation	More than 200 pages, including known bugs	Sparse	Detailed	Detailed
Online tutorial	Y	Y	Y	Y
Book	Y	N	N	Y
Years of development	>20	10	9	>20

*C, create a single neuron template, M, create a module and fit parameters to an experimental data, T, create a network, R, run a simulation, P, plot graphs. Y, Yes, N, No*.

All simulators have inter-user and user-developer communication mechanisms, through online forums or e-mail lists. The quality of online documentations is a critical parameter for a user. Although all simulators tend to maintain online documentation, the quality of this documentation is variable. Finally, all simulators have online tutorials for beginners and user guideline books. We did not find an independent book about the NEST and BRIAN simulators, but there are chapters in books that are available online.

To finish our comparison of simulator usage, we perform a study of the most popular model database, *Model DB* (Hines et al., 2004) (see Figure 1). A pool of models implemented on each software over the last 3 years (Figure 1C) indicates increasing community interest in the BRIAN simulator as well as a slight decrease in interest in GENESIS and NEST. However, NEURON has remained the most popular software over the last 3 years and also on average ever, since *Model DB* has been in existence (Figure 1A). We believe that such a dramatic difference between the usage of NEURON and other software cannot be explained only by the fact that *Model DB* is supported by the NEURON founders. Indeed “Model DB—independent” assessment of simulator usage by a number of published papers, which used each simulator (Citation index, Figure 1B; Table S2 in Supplementary Material) indicates the same arrangement in software popularity: NEURON > GENESIS > BRIAN > NEST. This result also supports the authors’ personal experience based on the analysis of posters at Society for Neuroscience meetings over the last 5 years that NEURON is the most popular simulator in the Neuroscience field. It also reflects the number of years taken for simulator development: BRIAN and NEST were under development for a duration that is half that of NEURON and GENESIS (Table 4). It thus appears that NEURON is the *de facto* standard for carrying out simulations of brain networks.

### 3.3 Case Studies

We performed two case studies to compare the code complexity and single-threaded performance of the studied simulators. We used the most extreme cases in the range of model types that can be simulated on the selected software. It should be noted that we did not write modules for NEURON and NEST but used developers’ code that comes as a part of the source code distribution. For BRIAN, we used examples from the BRIAN website to limit code complexity to that presented in the available documentation (see Materials and Methods for more details). Again we had to exclude GENESIS from our case studies, because we could not find any examples or modules for the LIF model or a way to organize a robust Python interface with GENESIS.

#### 3.3.1 Case Study 1: Classical Pyramidal InterNeuron Gamma (PING) Oscillations

In the first case study, we used a standard network of PING oscillations. The network is a simplified version of the (Brunel and Wang, 2003; Atallah and Scanziani, 2009) model. It consists of 4,000 excitatory and 1,000 inhibitory LIF neurons and 500 Poisson generators. Synaptic interactions are modeled by a simple instantaneous pulse, as described in Case Study 1 in the Materials and Methods section. This case study is favorable for simulators oriented toward network properties with the simplest neural and synaptic models.

**Figure 2:**
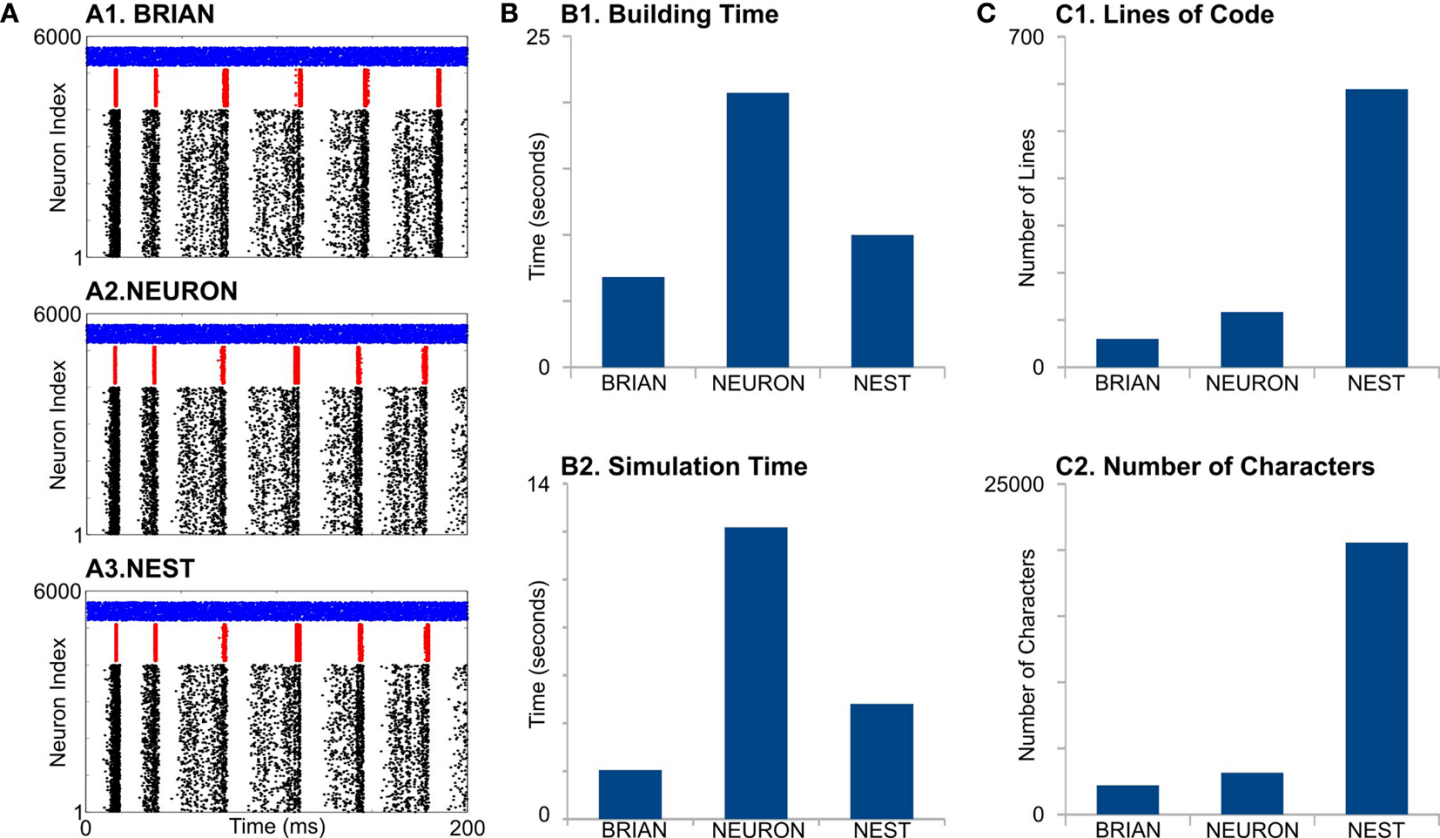
Case Study 1. **(A)** Raster diagrams of Standard Pyramidal—InterNeuron Gamma oscillations (PING) in network of 4,000 excitatory (**black dots**) and 1,000 inhibitory (**red dots**) leaky integrate-and-fire neurons and 500 Poisson spike generators (**blue dots**). Each dot is a spike of particular neuron within a population. First, 200 ms of 1 s run is shown. Diagrams were obtained from simulation on BRIAN (A1), NEURON (A2), and NEST (A3) software. **(B)** Evaluation of performance for studied software. B1 is a time required to build model, and B2 is a time required to simulate 1 s of model time. **(C)** Analysis of code complexity: number of lines (C1) and number of characters (C2) in modules and Python code for each software.

First, all simulators produce similar network activity (Figure 2A), although not identical. Note we constrain all the model parameters and random sequences to be exactly the same. The model building time was the longest for NEURON (Figure 2, B1), which is probably because that NEURON does not support batch connection creation; therefore, each connection has to be created independently. Indeed, this NEURON feature rises from the fact that for multicompartment models, connections cannot be represented just by indexes of pre- and post-synaptic neurons but also by the location of synapse on dendrite tree.

NEURON had the highest simulation time (Figure 2, B2). It is not surprising, because the network of LIF is not a favorable model for NEURON. Interestingly, BRIAN showed a better performance than the highly optimized low-level C++ NEST’s code.

Although neural and synaptic models are extremely simple in this case study, different simulators show dramatic differences in code complexity (Figure 2, C1,C2). In both lines of code (LOC) and number of characters (NOC), BRIAN uses the most concise language and shows best performance for this type of model. Indeed, the preference of NEST developers to use low-level C++ language leads to a very complicated code, which exceeds BRIAN and NEURON several times for both LOC and NOC counts. However, a recently introduced language for NEST modules, NESTML (Plotnikov et al., 2016), may significantly reduce both LOC and NOC in the future.

#### 3.3.2 Case Study 2: PostInhibitory Rebound InterNeuron Gamma (PIR-PING) Oscillations

In the second case study, we use PIR-ING network (Tikidji-Hamburyan et al., 2015) with a classical single-compartment, conductance-based Hodgkin–Huxley model (Hodgkin and Huxley, 1952). The network consists of 400 neurons with no external activation. Only inhibitory connections are presented in the model. An inhibitory synaptic current is described as a double-exponential dynamical process, which adds two differential equations to the main neuron dynamics. Note that we add a single-exponential excitatory synapse to each neuron in BRIAN and in the NEURON version of the model. We had to add this almost silent synapse to equalize the number of equations, which each package evaluates for each neuron, as the NEST module has two built-in synapses and modification of this module is not trivial. This case study is assumed to be favorable for simulators oriented toward accurate spatial-temporal integration, but it is still possible to implement it on “network-oriented” simulators.

**Figure 3:**
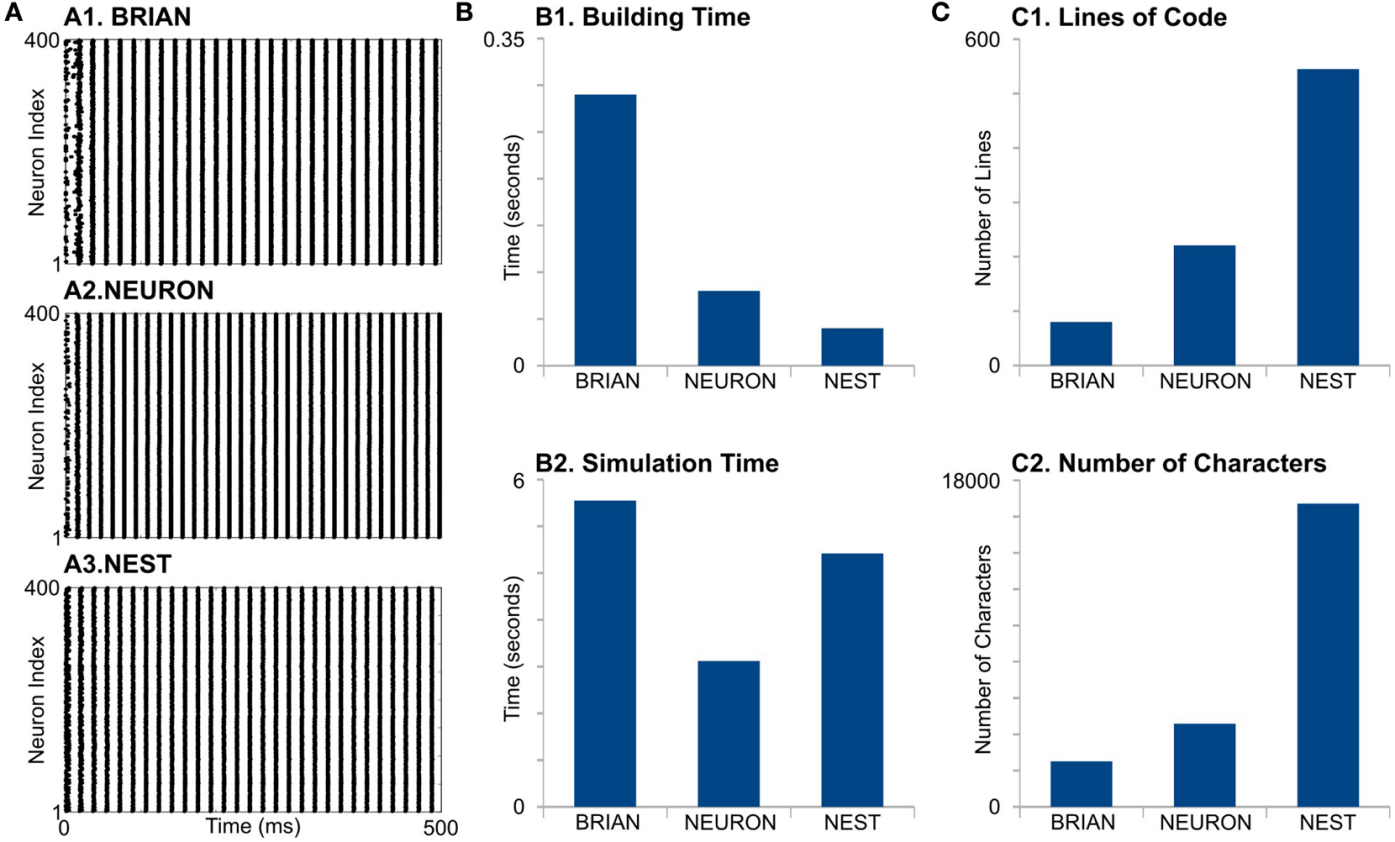
Case Study 2. **(A)** Raster diagrams of Standard PostInhibitory Rebound—InterNeuron Gamma oscillations (PIR-ING) in network of 400 Hodgkin–Huxley (**black dots**). Each dot is a spike of particular neuron within a population. Diagram was obtained from simulation on BRIAN (A1), NEURON (A2), and NEST (A3) software. Same analysis of performance **(B)** and code complexity **(C)** as in Figure 2.

First, we find that simulators also do not show identical results (Figure 3A). The differences appear in the initial network oscillations and in the frequencies of network oscillations. Note that random initialization as well as random connectivity were generated once; therefore, we constrain all the model parameters and random sequences to be exactly the same. We do not have valuable explanations for this difference and leave this question open for further research.

For model formation, BRIAN needs more time that any other simulator (Figure 3, B1). It probably indicates longer time for in-line compilation. The building time also did not take into account that NEST and NEURON need time for module compilation, which is not part of the script. However, note that NEST and NEURON need compilation only once, while BRIAN needs time for analysis and compilation for every model run. This may be one of the biggest disadvantages of BRIAN for big and complicated models. Surprisingly, NEURON does not show the fastest building time, even though it is mostly oriented to this kind of model. Again, the long formation time in NEURON is, probably, due to the absence of batch methods to create connections and each NetCon object has to be created independently in the script.

NEURON shows the best performance for this model type (Figure 3, B2). Note that off-the-shelf NEURON uses an exponential-Euler method to solve ion-channel gating variables and high-order numerical methods only for voltage. However, switching on different solvers does not reduce NEURON’s performance significantly (not shown); therefore, we assume that high NEURON performance is due to using tabulated results for the right-hand side of differential equations for the gating variables. Although BRIAN’s simulation time is the longest in this case study, it may be significantly improved if the less accurate “exponential Euler” is used.

Evaluation of code complexity shows that BRIAN has the most compact code, while the NEST code exceeds this more than three times in both LOC and NOC counters, although this may be improved with NESTML language. Again, as we mentioned earlier, the implementation of low-level languages does not guarantee optimization.

## 4 Discussion and Conclusion

### 4.1 Other Software Packages for Brain Network Simulations

We consider here the three most popular software packages for brain network simulations: BRIAN, GENESIS, and NEURON (in alphabetic order) in addition to the NEST simulator. However, there are many other less popular packages, which target the same or similar fields. For example, we did not consider here general-purpose software, such as XPP-auto (Ermentrout, 2002), the package for studying general dynamical systems, or MATLAB™, a general mathematical framework, due to an absence of specific routines for neural network simulation. Comparative analysis between these software and specialized simulators is hardly possible. The same can be said regarding models developed using general-purpose computer languages, such as C/C++, Fortran, and Python. Note that Python can be used as the language to describe a network structure as well as individual morphological detailed neurons. In this case, Python is not used as a main computational kernel but forms a wrapper around computationally intensive parts of the software.

Note that simulators that allow the study of effects of precise ion-channel positions on dendrite membranes (see for example, Cannon et al., 2010) or effects of diffusion of individual molecules in cytoplasm (see for example, Hepburn et al., 2012) are beyond the scope of this article, due to limited application of this software for large brain network simulations. We did not consider other simulators due to a low frequency of usage or early stage of software development (for example, NSC, Drewes, 2005, or NeuroCAD, Tikidji-Hamburyan and Markin, 2008). We also excluded from this analysis all simulators that can support only one neural/synaptic model or have a very limited range of supported models, for example, only single-compartment spike or rate-based models (ANNarchy—C++ code generator for software and hardware implementation, Vitay et al., 2015; Auryn—optimized for multiscale time resolution and formal representation of spike-dependent plasticity, Zenke and Gerstner, 2014; and GeNN—oriented on maximal performance on graphical processors, Yavuz et al., 2016).

### 4.2 Model Range and Selection of the Case Studies

As we stated earlier, we attempted to select the most “distal” model types in the model-type domain, which may be simulated on selected simulators. However, it should be mentioned that other cases may be selected from the same domain. For example, one can study a single-compartment model and fully reconstructed neuron on BRIAN, NEURON, and GENESIS with the exclusion of NEST. Although in our opinion, the model-type range is the most critical characteristic of the brain network simulators, one can study the quality of HPC utilization and compare pGENESIS, NEURON, and NEST with the exclusion of BRIAN. We hope that our results will open a broad discussion in computational neuroscience and computer science communities and will trigger further independent wide-ranging studies of simulators in the computational neuroscience field.

### 4.3 Available Solvers

Although we consider here the performance of all packages “off-the-shelf”, at least NEURON and BRIAN offer a range of “solvers” for numerical integration of differential equations. For example, one such solver is CVODE (Hindmarsh et al., 2005) that can significantly increase the speed of simulations, especially when the spike activity is periodical with high synchronization. Indeed, this ability can help the user to use the most optimal solver for a specific model. However, a choice of the solver may not be a trivial problem. Therefore, a comparative study of performance can depend upon the choice of solver. For example, a linear Euler method may be 10-fold faster than the GNU Scientific Library implementation of Runge–Kutta 4/5 method (Vitay and Hamker, 2015). Indeed, NEURON and NEST modules were developed for specific models and, therefore, they are “aware” of the model type and change solvers accordingly (exponential Euler for LIF vs RK45 or modified Crank–Nicholson method for conductance-based models). Thus, the default solver is a reasonable choice at least. In contrast, BRIAN’s online documentation recommends the use of a “linear solver” with the suggestion to try other options if BRIAN fails to generate code for this solver. However, when the recommended solver fails, the user has to choose a solver from the list. It seems that the “off-the-shelf” solvers are model specific and well optimized, but we leave this question outside of the scope of this study.

Another critical question, which we also left outside of the scope of this study and just briefly discuss here, is how simple is it to change a solver in a model. Different simulators offer different functionalities. In NEURON and BRIAN, the embedded solvers may be changed by editing one parameter in a script or in a module. In NEST, changing the solver may not be a trivial problem, due to the use of low-level C++ language for module development. For example, if the user wants to implement the CVODE solver, which requires that all dynamical variables must be held in one vector, she or he has to modify not only the module code but also the NEST kernel code, due to the inner object-oriented class-based architecture. In NEURON and BRIAN, a similar problem appears if the user wants to use a solver, which is not embedded into the simulator (for example, use CVODE in BRIAN).

### 4.4 BRIAN Performance for Different Code Generation Modes

As we noted in Section 2, we used BRIAN in “default” code generation mode, i.e., numpy code. However, the user can switch to Cython or pure C-code generation to improve performance. Indeed, Cython can speed up computation by at least 20–30% in both case studies. Moreover, depending on the hardware and installed software, a 4- to 5-fold increase may be achieved. However, the first run (or run after any changes in the scripts) will take at least 3 times as long as any subsequent numpy code, due to the longer process of compilation. Therefore, in the development stages, numpy is the preferred code generator, while long runs of a well debugged model will perform better using the Cython code generator, with modules precompiled through a brief run.

BRIAN also can generate so-called “Standalone code,” which can use OpenMP threading. Standalone code may potentially be “much faster” than numpy/Cython code but with additional limitations on functionality. It seems that the OpenMP code generator is still in development and “may be not accurate” according to Brian2 documentation.

### 4.5 High-Performance Computing

With the current development of computational clusters and multicore computers, the ability of a simulator to utilize the power of HPC is critical, in order to enable simulations of large scale networks. Moreover, it is critical to have a simple way for mapping existing models of brain networks on clusters or multicore computers. It should be noted that HPC computation is one of the weakest characteristics of BRIAN software. BRIAN does not support MPI on clusters, and stand-alone OpenMP and GPU versions have limited functionality. Using NEURON, users have to modify scripts if the model was developed without using the *ParallelContext* mechanisms, and later, it has to be mapped onto the cluster. This requires additional effort and may generate additional bugs. However, this approach is flexible and allows the user to map the neural network architecture onto cluster hardware with a better balance, which may be critical for both heterogeneous network architectures and heterogeneous clusters. It is only when the user wants to use multi-threading that NEURON’s script requires minimal changes. NEST offers the simplest way to map a model onto a cluster or multicore computers without any changes in scripts. That is definitely the strongest advantage of NEST.

### 4.6 Usability

It is important to note that front-ends, such as PyNN and neuroConstruct, as well as high-level structural languages, such as NeuroML, may help hide the code complexity at the simulator level. Such front-ends can help to decrease both LOC and NOC or totally/partially substitute code development by manipulations with user-friendly GUI. However, front-ends use internal converters to generate final script(s) for a specific simulator based on model description. The development of such converters is more complicated for simulators with high NOC and LOC (see Figures 2C and 3C), than for concise languages. Therefore, our analysis of code complexity can be considered not only as an estimation of the amount of effort required for model development but also as a potential barrier for developing a converter. Although the code conciseness does not guarantee readability or comprehension, a better analysis is not possible due to differences in languages for the modules used by the simulators.

While the authors acknowledge that the number of lines of code may not be fully accurate in capturing the software development effort, it remains a valuable quantitative metric for comparing codes developed in high-level languages and software frameworks. Notably, DARPA’s High Productivity Computing Systems (HPCS) program that ran during the first decade of the 21st century used the Source Lines of Code (SLOC) metric, which is the same (Dongarra et al., 2008). SLOC was used in comparing the productivity of different parallel computing languages. Furthermore, Lind and Vairavan (1989) carried out an experimental investigation of software metrics and concluded that conceptually simple measures such as LOC exhibit a high level of correlation to the development effort, equaling or surpassing other software metrics. In our study, we augment the LOC metric with another simple metric used by Lind and Vairavan (1989), the number of characters (NOC), as we did in a prior study in parallel computing (Cantonnet et al., 2004).

### 4.7 Summary

Here, we considered the most popular software packages for brain network simulations: BRIAN, GENESIS, NEST, and NEURON. All simulators tend to support a large range of models of neurons and synapses. However, the performance of these simulators is different, and the complexity of codes required for model description is diverse. Not all simulators can be used on computational clusters, but all of them may be used on multicore computers with some limitations.

## Author Notes

The authors have more than 10 years of experience in spiking neural network simulations as well as a history of developing brain network simulators, NeuroCAD (Tikidji-Hamburyan and Markin, 2008). We routinely use NEURON, BRIAN, XPP, and NEST. However, we should admit that we have limited experience in using GENESIS and pGENESIS. Our evaluation of (p)GENESIS is mostly based upon the documentation and available examples.

## Author Contributions

RT-H, ZB, and TE-G designed the research; RT-H conducted the research; RT-H, VN, and TE-G discussed the results; RT-H and VN wrote the manuscript.

## Conflict of Interest Statement

The authors declare that the research was conducted in the absence of any commercial or financial relationships that could be construed as a potential conflict of interest.

## Footnotes

^1^http://www.nest-simulator.org/py_sample/multi-compartment-neuron-example.
