# Corrigendum: Software for Brain Network Simulations: A Comparative Study

**DOI:** 10.3389/fninf.2018.00022

**Published:** 2018-05-01

**Authors:** Ruben A. Tikidji-Hamburyan, Vikram Narayana, Zeki Bozkus, Tarek A. El-Ghazawi

**Affiliations:** ^1^School of Engineering and Applied Science, George Washington University, Washington, DC, United States; ^2^Computer Engineering Department, Kadir Has University, Istanbul, Turkey

**Keywords:** computational neuroscience, brain network simulators, spiking neural networks, comparative study, phenomenological model, conductance-based model

We found an inaccuracy in the benchmark test for the NEST simulator in Case Study 2 of the article. The module we used does not completely implement the system of differential Equation (2) in the article. Instead of a synaptic model with reversal potentials, it implements a synaptic model with an injected current. This slightly reduces the computational load for the NEST benchmark. We believe that the module hh_psc_alpha is the closest to the system (2) module of modules distributed with NEST.

We are confident that this inaccuracy does not qualitatively affect the results or the scientific conclusions of the article in any way.

Also, we would like to mention that the correct citation for NEST 2.12.0 is: Kunkel, Susanne; Morrison, Abigail; Weidel, Philipp; Eppler, Jochen Martin; Sinha, Ankur; Schenck, Wolfram; Schmidt, Maximilian; Vennemo, Stine Brekke; Jordan, Jakob; Peyser, Alexander; Plotnikov, Dimitri; Graber, Steffen; Fardet, Tanguy;Terhorst, Dennis; Mørk, Håkon; Trensch, Guido; Seeholzer, Alex; Deepu, Rajalekshmi; Hahne, Jan; Blundell, Inga; Ippen, Tammo; Schuecker, Jannis; Bos, Hannah; Diaz, Sandra; Hagen, Espen; Mahmoudian, Sepehr; Bachmann, Claudia; Lepperød, Mikkel Elle; Breitwieser, Oliver; Golosio, Bruno; Rothe, Hendrik; Setareh, Hesam; Djurfeldt, Mikael; Schumann, Till; Shusharin, Alexey; Garrido, Jesús; Muller, Eilif Benjamin; Rao, Arjun; Vieites, Juan Hernando; Plesser, Hans Ekkehard (2017, March 1). NEST 2.12.0. Zenodo. http://doi.org/10.5281/zenodo.259534.

## Author contributions

RT-H found and corrected inaccuracy. RT-H submitted the corrigendum on behalf of all authors.

### Conflict of interest statement

The authors declare that the research was conducted in the absence of any commercial or financial relationships that could be construed as a potential conflict of interest.

