# Supplementary material for: Software for Brain Network Simulations: A Comparative Study
Source: Front Neuroinform. 2017 Jul 20;11:46. doi: 10.3389/fninf.2017.00046 (PMC5517781; doi:10.3389/fninf.2017.00046)
Supplement: Supplementary file 1 [file Data_Sheet_1.PDF]

Supplementary Material:
Software for Brain Network Simulations: A Comparative Study

Ruben A. Tikidji-Hamburyan [1,*], Vikram Narayana [1], Zeki Bozkus [2], and Tarek A. El-Ghazawi [1]

*Correspondence:
Ruben A. Tikidji-Hamburyan
rath@gwu.edu

Supplementary Table 1. Number of records on Model DB for different simulators at August 2016

1	AnimatLab v1	2
2	BioPAX	1
3	Brian	37
4	Catacomb	1
5	CellExcite	1
6	CellML	1
7	Chemesis	2
8	CNrun	1
9	COMSOL	2
10	CONTENT	2
11	COPASI	1
12	CSIM	4
13	EDLUT	2
14	Emergent/PDP++	3
15	ERNST (Event Related Neuronal Simulation Tool)	1
16	FEMLAB - COMSOL	0
17	GENESIS	40
18	GNUstep NeXTStep/OpenStep	1
19	IChMASCOT	0
20	IDL	1
21	IGOR Pro	3
22	IonChannelLab	1
23	KInNeSS	2
24	L-Neuron	0
25	LCG	1
26	LFPy	0
27	LibRoadRunner	1
28	MadSim	2
29	MCell	2
30	MOOSE/PyMOOSE	2
31	MVASpike	1
32	Nengo	2
33	Neosim	0
34	NEST (formerly BLISS/SYNOD)	7
35	NetPyNE	1
36	Network	1
37	NeuGen	0
38	neuroConstruct	6
39	NeuroML	5
40	NEURON	558
41	NeuronC	0
42	NeuronetExperimenter	1
43	NEURONPM	2
44	Neuronvisio	1
45	NeuroRD	3
46	parplex	2
47	PCSIM	1
48	PSICS	1
49	PSpice	2
50	PyNN	2
51	QuB	1
52	ReMoto	1
53	SABER	1
54	SBML	4
56	SNNAP	21
57	Snnet	0
58	Sspice Symbolic SPICE	1
59	Surf-Hippo	0
60	Synthesis	0
61	Topographica	2
62	VERTEX	1
63	Virtual Cell	3
64	XML	4
65	XNBC	0
66	Yale HMM	1

Supplementary Table 2. Citation index

For NEST and NEURON, numbers of citations were obtained from the official websites. The citation lists were truncated by the end of 2016. The number of citations for GENESIS was obtained through Google scholar website as a number of articles cited the chapter "Constructing new models", JM Bower - The Book of GENESIS, 1998 - Springer. The number of citations for Brian was obtained through Google scholar website as a number of articles cited two BRIAN introductory articles, officially recommended for citations: Goodman D and Brette R (2008) Brian: a simulator for spiking neural networks in Python. Front. Neuroinform. doi:10.3389/neuro.11.005.2008 and Goodman DFM and Brette R (2009). The Brian simulator. Front Neurosci doi:10.3389/neuro.01.026.2009. For GENESIS and BRIAN the numbers of citations were collected on April 8th , 2017.

BRIAN	541
GENESIS	1163
NEST	350
NEURON	1840

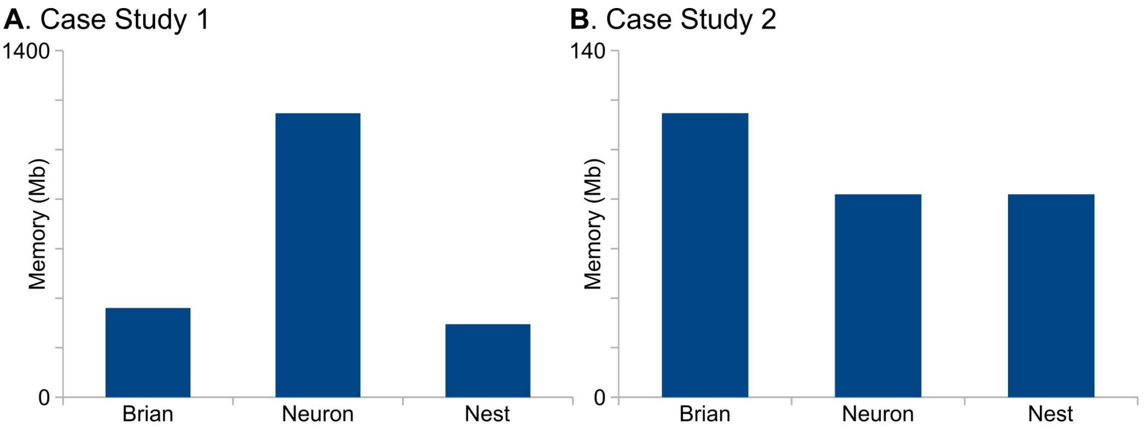

Supplementary Figure 1. Memory usage for Case study 1(left) and 2(right) based on the output of the top command
